# *ALDH2* Genotype Has No Effect on Salivary Acetaldehyde without the Presence of Ethanol in the Systemic Circulation

**DOI:** 10.1371/journal.pone.0074418

**Published:** 2013-09-13

**Authors:** Andreas Helminen, Satu Väkeväinen, Mikko Salaspuro

**Affiliations:** Research Unit on Acetaldehyde and Cancer, Faculty of Medicine, University of Helsinki, Helsinki, Finland; National Cancer Center, Japan

## Abstract

**Background:**

Acetaldehyde associated with alcoholic beverages was recently classified as carcinogenic (Group 1) to humans based on uniform epidemiological and biochemical evidence. ALDH2 (aldehyde dehydrogenase 2) deficient alcohol consumers are exposed to high concentrations of salivary acetaldehyde and have an increased risk of upper digestive tract cancer. However, this interaction is not seen among ALDH2 deficient non-drinkers or rare drinkers, regardless of their smoking status or consumption of edibles containing ethanol or acetaldehyde. Therefore, the aim of this study was to examine the effect of the *ALDH2* genotype on the exposure to locally formed acetaldehyde via the saliva without ethanol ingestion.

**Methods:**

The *ALDH2* genotypes of 17 subjects were determined by PCR-RFLP. The subjects rinsed out their mouths with 5 ml of 40 vol% alcohol for 5 seconds. Salivary ethanol and acetaldehyde levels were measured by gas chromatography.

**Results:**

Acetaldehyde reached mutagenic levels rapidly and the exposure continued for up to 20 minutes. The mean salivary acetaldehyde concentrations did not differ between *ALDH2* genotypes.

**Conclusions:**

For ALDH2 deficient subjects, an elevated exposure to endogenously formed acetaldehyde requires the presence of ethanol in the systemic circulation.

**Impact:**

Our findings provide a logical explanation for how there is an increased incidence of upper digestive tract cancers among ALDH2 deficient alcohol drinkers, but not among those ALDH2 deficient subjects who are locally exposed to acetaldehyde without bloodborne ethanol being delivered to the saliva. Thus, ALDH2 deficient alcohol drinkers provide a human model for increased local exposure to acetaldehyde derived from the salivary glands.

## Introduction

Cancers of the upper digestive tract are often found at an advanced stage, remain difficult to treat and have a high mortality rate. The risk for upper digestive tract cancer for alcoholics who have an impaired ability to eliminate acetaldehyde due to a single point mutation in the mitochondrial aldehyde dehydrogenase 2 (*ALDH2*) gene is 10-fold that for alcoholics without the mutation [1,2]. The substantially increased risk of esophageal squamous cell carcinoma (ESCC) for ALDH2 deficient alcoholics has provided the most persuasive evidence for the carcinogenic potential of acetaldehyde in humans [3-10].

ALDH2 is a low *K*_*m*_ mitochondrial enzyme that oxidizes acetaldehyde to acetate. Approximately 30-50 percent of Eastern Asians carry an allele (ALDH2*2) of the *ALDH2* gene, which contains a single nucleotide polymorphism (SNP) and results in the synthesis of an inactive ALDH2 enzyme [11]. In addition to an increased risk of upper digestive tract cancer, ALDH2 deficiency is also associated with an increased exposure of the upper digestive tract mucosa to salivary acetaldehyde from drinking alcohol [12-14]. Uniform interaction between ALDH2*2 genotype, alcohol consumption and upper digestive tract cancer risk found in epidemiological data and the elevated local acetaldehyde exposure of ALDH2*2 carriers who consume alcohol provide convincing evidence for the specific carcinogenic potential of acetaldehyde in the pathogenesis of upper digestive tract cancers. Based on these findings, the International Agency for Research on Cancer (IARC) recently classified acetaldehyde associated with alcoholic beverages i.e. present in alcoholic beverages and/or formed endogenously from ethanol to be a Group 1 carcinogen in humans [15].

In addition to alcohol consumption, smoking is also a generally accepted major etiological factor for upper digestive tract cancers [16]. The multiplicative and dose-dependent effects of alcohol and tobacco on ESCC risk have been known for decades and confirmed in studies and meta-analyses [17,18]. However, it was recently shown that the risk of ESCC for non-drinking ALDH2 deficient smokers was not higher than that for non-drinking smokers with functional ALDH2 enzyme [8].

Unlike the case for alcohol ingestion, in which the *ALDH2* genotype significantly potentiates the risk of upper digestive tract cancer, there seems to be no apparent increased ESCC risk with ALDH2 deficient non-drinkers [7,19]. Nevertheless, they may use edibles that contain low amounts of acetaldehyde or ethanol, e.g. pickled food [20]. This suggests that the amounts of acetaldehyde formed locally in the oral cavity from ethanol or delivered to saliva either from tobacco smoke or food may be independent of the *ALDH2* genotype and raises the question about whether the *ALDH2* genotype only has an effect when systemic ethanol is available.

Our present study follows up on our earlier paper in which 7 ALDH2 deficient individuals and 13 individuals with the functional ALDH2 enzyme ingested 0.5 g/kg of ethanol and their salivary acetaldehyde levels were followed thereafter at 20 minute intervals for a period of 240 minutes [12]. At each time point, salivary acetaldehyde concentrations of ALDH2 deficient individuals were two to three times higher than those of individuals with the functional ALDH2 enzyme (p<001). In the present study, our objective was to examine the effect of the *ALDH2* genotype on the exposure of the upper digestive tract to salivary acetaldehyde when ethanol is only rinsed in the mouth, but not ingested.

## Materials and Methods

### Study subjects

20 healthy Eastern Asian volunteers (10 female, 10 male) were recruited into the study. The mean age of the participants was 25.7 years (range: 21-38 years) and the mean body mass index (BMI) 20.9 (range: 18.0-27.8). All the participants were of Chinese origin and moderate alcohol drinkers i.e. they consumed <20 drinks/week (men) or <14 drinks/week (women). Half of the volunteers reported having a history of alcohol-related flushing symptoms, which are known to correlate significantly with the ALDH2*2 genotype [21]. Exclusion criteria were pregnancy and use of antimicrobial medication within 30 days prior to the study visit. The results of three participants were omitted from the study: in two cases genotypes could not be analyzed and the third participant was omitted because of insufficient salivary secretion for analysis.

### Ethics Statement

The study was approved by the co-ordinating Ethics Committee, Hospital District of Helsinki and Uusimaa (Finland). Signed informed consent to participate in the study was obtained from each study participant.

### Study design

The participants were instructed to abstain from drinking alcohol for 24 hours before the study visit day and to fast for two hours before giving samples. Smoking was prohibited on the day of the study. Salivary samples which were used for genotyping were collected prior to the ethanol rinsing experiment. For the rinsing procedure the volunteers gave a baseline salivary sample and then rinsed their mouths with 5 ml of 40 vol% alcohol for 5 seconds, after which the oral contents were discharged and salivary samples were collected at 30 s, 2 min, 5 min, 10 min, 15 min and 20 min after discharging. The participants also answered a questionnaire regarding their oral health, alcohol use, smoking, diet and medication.

### Genotyping

The genotyping protocol used was modified from Hayashida et al. [22]. Whole saliva was collected from each participant and stored at -20°C until analysis. The samples were analyzed using direct polymerase chain reaction restriction fragment length polymorphism (PCR-RFLP) on a Mastercycler Gradient (Eppendorf) to identify a SNP of the *ALDH2* gene (rs671). The PCR protocol included one cycle of 95°C for 5 min, 40 cycles of 98°C for 10 s, 60°C for 30 s, and 74°C for 45 s and a final cycle of 74°C for 2 min. A 430-bp DNA fragment that contained the polymorphic site of *ALDH2* was amplified by PCR using the forward primer 5′-TCAAATTACAGGGTCAACTGCT-3′ and the reverse primer 5′-GGCTGGGTCTTTACCCTCTC-3′ (Sigma-Aldrich). The PCR reaction required 7.5 µl of distilled water, 12.5 µl of 2X Xtreme Buffer, 2.5 µl of 2 mM deoxyribonucleotide triphosphates, 10 pmol each for the two *ALDH2* primers, and 0.5 U of KOD Xtreme DNA polymerase (1 U/µl, KOD Xtreme Hot Start DNA Polymerase, Novagen) in a total volume of 25 µl. PCR products were digested using *Acu*I according to the manufacturer’s instructions (New England Biolabs Inc.). The 430 bp ALDH2*1 fragment was cut into two fragments of 296 and 134 bp. The ALDH2*2 allele (2*/2*) was not cut. Fragments were analyzed by using gel electrophoresis on a 2% agarose gel. Samples of five randomly selected participants were analyzed twice to assess the reliability of the genotyping protocol.

### Measurement of salivary acetaldehyde and ethanol

50 µl of 6 M perchloric acid was added to 450 µl of saliva to stop organic reactions, after which the samples were immediately sealed in 20 ml vials and stored at -20°C until analysis. Dual or triple parallel samples were collected at each time point whenever possible to confirm analytical reliability. Acetaldehyde and ethanol concentrations were measured by headspace gas chromatography as previously described [23]. 100 µM acetaldehyde samples were processed with each batch of study samples as controls.

### Statistical analysis

Target sample size (6) was calculated for an effect size of 1.5 SD with an alpha level of 0.05 and a power of 0.80 using data from our previous study with ALDH2 deficient subjects [12]. Interactions between different genotypes and salivary acetaldehyde concentration were estimated by using repeated measures ANOVA with Huynh-Feldt corrections. The means of the samples were compared using the Mann-Whitney U test. The means of parallel duplicate and triplicate samples were used when determining acetaldehyde concentrations. Group means were used when calculations included a missing data point. All calculations were made by using SPSS 15.0.1 (SPSS Inc., Chicago, IL) statistical software.

## Results

### Health questionnaire

All of the participants reported brushing their teeth at least twice per day, three participants (15%) also used mouthwashes. Nine participants (45%) were smokers and all reported consuming <20 alcohol drinks/week (men) or <14 alcohol drinks/week (women). Of participants with the ALDH2*2 genotype, four (67%) were smokers. Of the participants without the ALDH2*2 genotype, four (36%) were smokers. The genotyping of one smoker was unsuccessful. No chronic illnesses were reported. Aside from one participant using oral medication for birth control, no regular medications were reported. Antibiotics had not been used for at least 30 days. Two participants (10%) reported following a non-lactose diet. One participant (5%) followed a vegetarian diet. Only one participant (5%) reported consuming products that contain lactic acid bacteria.

### Genotyping

**Figure 1:**
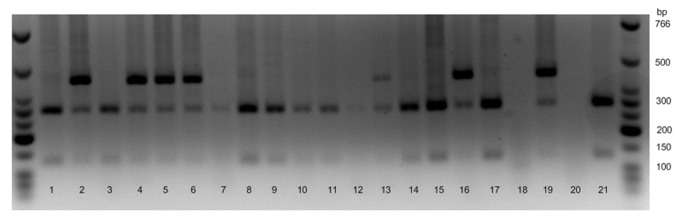
Gel electrophoresis of digested ALDH2 fragments. Lane 21 is a positive PCR control, lane 20 is a negative control. Lanes 2, 4-6, 13, 16 and 19 are ALDH2*1/*2. Lanes 1, 3, 7-12, 14-15 and 17 are ALDH2*1/*1. Lane 18 is unspecified.

Eleven participants carried the ALDH2*1/*1 genotype, five the ALDH2*1/*2 genotype and one participant the ALDH2*2/*2 genotype (Figure 1). The samples of two participants could not be analyzed. One other participant produced insufficient saliva for acetaldehyde analysis and was not also genotyped. The results of five randomly selected samples were analyzed as a replicate for reliability assurance and subsequently matched with the original analyses.

### Salivary acetaldehyde concentration

**Figure 2:**
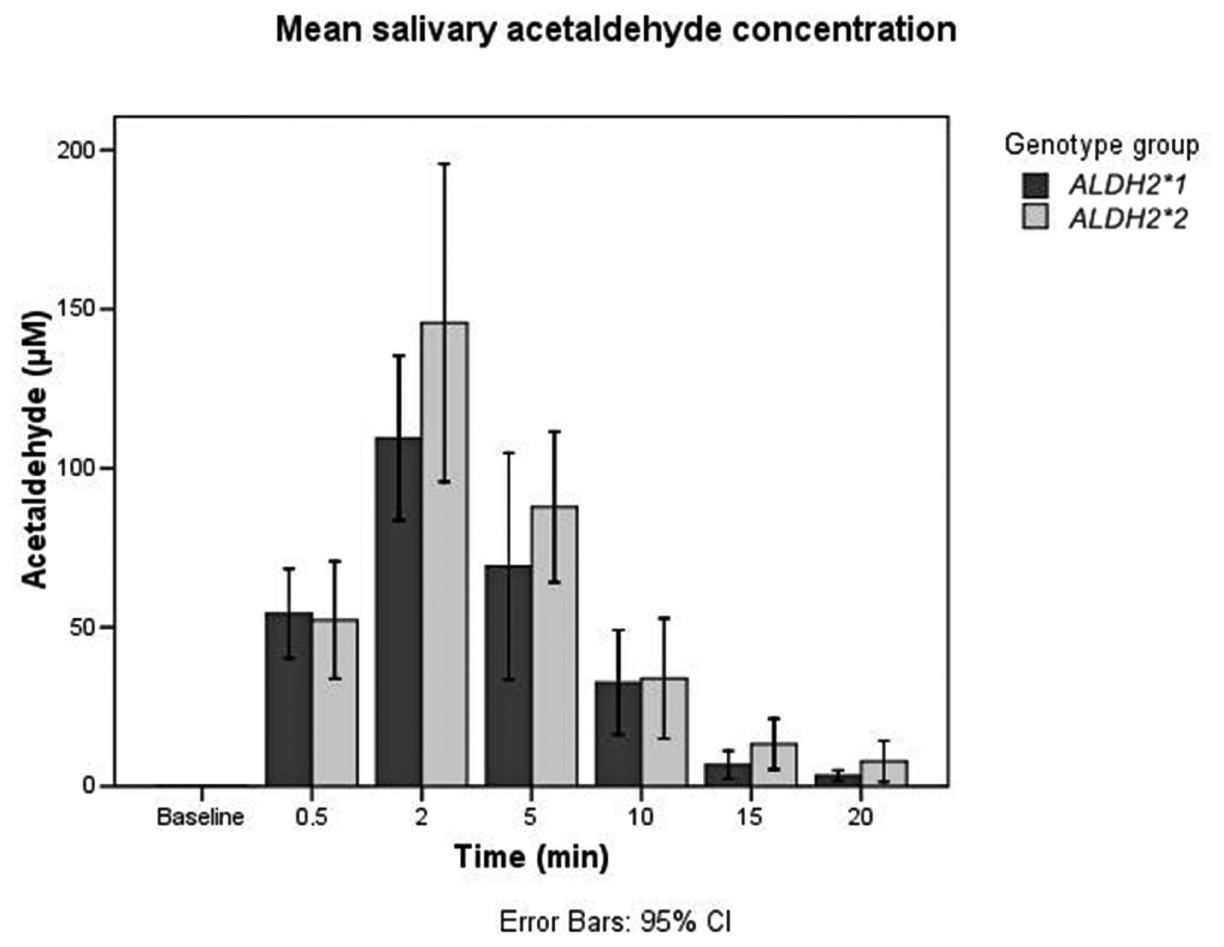
Mean salivary acetaldehyde concentration before and after ethanol exposure according to genotype group. ALDH2*1 group: ALDH2*1/*1 (n=11). ALDH2*2 group: ALDH2*1/*2 (n=5) and ALDH2*2/*2 (n=1).

After the 5 s of oral exposure to 40 vol% alcohol, salivary acetaldehyde concentrations rose quickly and peaked at 2 min (Figure 2, Table 1). Detectable amounts of acetaldehyde were found for up to 20 min after the ethanol exposure. A comparison between subjects with the active ALDH2*1 allele (ALDH2*1/*1, n=11) and those with the deficient ALDH2*2 allele (ALDH2*1/*2, n=5 and ALDH2*2/*2, n=1) found that there were no statistically significant differences in the mean *in vivo* acetaldehyde levels of the saliva samples for any of the time points (Table 1, Figure 2). The interactions between different *ALDH2* genotypes and salivary acetaldehyde concentration were not statistically significant (F=1.482, p=0.237). Also, analysis of the area under the curve didn’t show a statistically significant difference between the genotype groups (Mann-Whitney U 48, p=0.149). There were no statistically significant differences in levels of salivary acetaldehyde between male and female subjects or between *ALDH2* genotype variants. Also, the interaction between smoking and salivary acetaldehyde concentration was not statistically significant (F=0.562, p=0.620).

**Table 1:** Mean salivary acetaldehyde concentration according to genotype group (μM ± SD).

**Timepoint**	ALDH2*1 (n=11)	ALDH2*2 (n=6)	**Mann-Whitney U-test**	**P**
0.5 min	54.3 ± 21.0	52.3 ± 17.6	31	0.89
2 min	109.5 ± 38.5	145.8 ± 47.2	14	0.06
5 min	69.2 ± 52.9	87.9 ± 22.6	16	0.10
10 min	32.7 ± 24.5	33.9 ± 18.0	27	0.59
15 min	6.8 ± 6.6	13.3 ± 7.6	18	0.15
20 min	3.3 ± 2.5	7.8 ± 6.1	17	0.10

### Salivary ethanol concentration

Measured ethanol levels peaked at 30 s and thereafter declined rapidly (Table 2). Detectable amounts of ethanol were found for up to 20 min after initial exposure. A comparison between subjects with the active ALDH2*1 allele (ALDH2*1/*1, n=11) and those with the deficient ALDH2*2 allele (ALDH2*1/*2, n=5 and ALDH2*2/*2, n=1) found that there were no statistically significant differences in the mean *in vivo* ethanol levels of the saliva samples for any of the time points (Table 2). The interactions between different *ALDH2* genotypes and salivary ethanol concentration were not statistically significant (F=1.133, p=0.316). There were no statistically significant differences in levels of salivary ethanol between male and female subjects or between *ALDH2* genotype variants. Also, the interaction between smoking and salivary ethanol concentration was not statistically significant (F=1.534, p=0.237).

**Table 2:** Mean salivary ethanol concentration according to genotype group (mM ± SD).

**Timepoint**	ALDH2*1 (n=11)	ALDH2*2 (n=6)	**Mann-Whitney U-test**	**p**
0.5 min	690.1 ± 297.5	899.6 ± 508.3	27	0.59
2 min	248.1 ± 117.7	341.4 ± 185.5	25	0.46
5 min	82.0 ± 54.6	124.0 ± 82.4	22	0.30
10 min	25.1 ± 24.6	28.6 ± 20.3	32	0.96
15 min	4.5 ± 6.2	6.8 ± 5.1	28	0.66
20 min	1.1 ± 1.6	2.6 ± 2.8	24	0.39

## Discussion

We have earlier demonstrated that after an oral ingestion of a moderate dose (0.5 g/kg) of ethanol, salivary acetaldehyde levels measured at 20 minute intervals are 2-3 times higher among ALDH2 deficient individuals (n = 7) than in those with functional ALDH2 enzyme (n = 13) [12]. In that study, a statistically highly significant (p<0.001) difference lasted for 4 hours without overlapping SEM values at any time point. The association between ingested ethanol and an elevated concentration of salivary acetaldehyde among ALDH2 deficient individuals has been confirmed in two later studies [13,14].

Our present results show for the first time that after a brief oral exposure to non-ingested ethanol, the concentration of salivary acetaldehyde of ALDH2 deficient subjects is not significantly higher than that of subjects with normal ALDH2 activity. It should be noted, however, that at the 2 min time point, means of salivary acetaldehyde between the groups showed a near-significant difference (p=0.06) which may become significant should the number of study subjects be considerably higher. At high concentrations of salivary ethanol (mean ranging from 248 to 899 mM) that are seen at the 2 and 5 min timepoints, enzymatic activity of the small salivary glands located in the oral mucosa may also contribute to our findings. At the 10 and 15 min time points salivary ethanol concentrations had decreased to 5-30 mM, a level that is comparable to those found after oral ingestion of alcohol [12].

No measurable levels of acetaldehyde or ethanol were found from the baseline salivary samples taken before subjects rinsed their mouths with 40 vol% alcohol. This is in accordance with earlier findings showing that without the presence of ethanol or tobacco smoke, normal saliva does not contain measurable levels of acetaldehyde [23,24]. The rapid rate of acetaldehyde production at the high initial concentrations of salivary ethanol can be explained by the presence of high *K*_m_ ADH enzymes in the gingiva and the lingual mucosa [25] and also by the fact that microbial ADH enzymes are not fully saturated at lower ethanol concentrations [23]. In concordance with our earlier findings, measurable amounts of ethanol and acetaldehyde could be found in the saliva for up to 20 min after rinsing of the mouth with a strong alcohol solution [26].

We used direct PCR in our *ALDH2* genotyping protocol in order to decrease the risk of sample contamination. Our study did not include the analysis of other genetic factors involved in ethanol metabolism, such as the *ADH1B* genotype. This warrants further studies that should focus on the possible role of *ADH* polymorphisms in the exposure of the oral cavity to ethanol without its ingestion.

Acetaldehyde is widely present in the environment and has been found to be mutagenic and carcinogenic in vitro and in animal experiments [27-29]. A recent study demonstrated that N2-ethylideoxyguanosine adducts, which are mutagenic DNA adducts previously linked to acetaldehyde exposure, are also found in the human oral cavity after drinking alcohol [30].

Acetaldehyde in concentrations of 100 µM and above has been shown to result in an exponential increase in mutagenic DNA lesions [31]. Acetaldehyde levels of this magnitude can be found in saliva both during and after an alcohol challenge in addition to during active tobacco smoking [23,24,26]. For individuals who have normal ALDH2 activity, this is by and large due to the bacteria and yeasts present in normal oral microflora that are able to oxidize ethanol to acetaldehyde, but their capacity to further oxidize acetaldehyde to acetate is limited [13,23,32-34]. Thus, the presence of ethanol in saliva leads to the accumulation of salivary acetaldehyde in mutagenic concentrations both in vitro and in vivo. The lack of low *K*_m_ ALDH enzymes and the presence of high *K*_m_ ADH enzymes in the gingiva and the lingual mucosa may further increase the local exposure to carcinogenic concentrations of salivary acetaldehyde [25].

The official criterion for alcoholic beverages is that they contain 2.8 vol% or more of alcohol and their consumption is systematically followed and used in cancer epidemiology. However, many non-alcoholic beverages and edibles produced by fermentation processes may contain low but significant levels of ethanol in addition to mutagenic concentrations of acetaldehyde [20,35,36]. Thus, consumption of these products has been suggested to cumulatively increase the exposure of the upper digestive tract to carcinogenic acetaldehyde [35,37-39]. This hypothesis is supported by epidemiological findings indicating that use of pickled food is a significant risk factor of esophageal and stomach cancer especially in East Asian countries [40,41]. Furthermore, positive *H. pylori* status combined with high consumption of pickled food has recently been shown to result in a 27-fold risk of noncardia gastric cancer [42]. Thus fermented foods and beverages, potential sources for local acetaldehyde exposure in the upper digestive tract, constitute a confounding factor that so far has not been widely considered in cancer epidemiology.

When combined, these results imply that the presence of ethanol in the systemic circulation is a key factor for the increased exposure of the upper digestive tract mucosa to endogenously formed acetaldehyde encountered with ALDH2 deficient consumers of alcohol. Likewise, these findings provide a logical explanation for the epidemiological findings that show that ALDH2 deficiency increases the risk of upper digestive tract cancer for alcohol drinkers, but not for non-drinkers who are exposed to acetaldehyde that is derived from sources that do not associate with the presence of ethanol in the systemic blood circulation [8,19]. Such sources of exogenous acetaldehyde can be food or tobacco smoke [20,24].

In conclusion, our present study supports earlier findings that show that the elevated levels of carcinogenic acetaldehyde found in the saliva of ALDH2 deficient individuals appear to be derived from the parotid glands [12,13], which produce acetaldehyde from systemic ethanol but are unable to detoxify it. ALDH2 deficiency does not increase salivary acetaldehyde levels, unless systemic ethanol is available. This finding helps to explain why the risk of upper digestive tract cancer is only increased for alcohol consuming ALDH2*2 carriers, but not for ALDH2 deficient non-drinkers and rare drinkers, regardless of their smoking status and possible consumption of edibles that contain ethanol or acetaldehyde. Thus, ALDH2 deficient alcohol drinkers provide a human model for increased local exposure to acetaldehyde derived from the salivary glands every time when they are drinking alcoholic beverages.
